# Learning to automate cryo-electron microscopy data collection with *Ptolemy*

**DOI:** 10.1107/S2052252522010612

**Published:** 2023-01-01

**Authors:** Paul T. Kim, Alex J. Noble, Anchi Cheng, Tristan Bepler

**Affiliations:** a Simons Machine Learning Center, Simons Electron Microscopy Center, New York Structural Biology Center, New York, NY USA; b Simons Electron Microscopy Center, New York Structural Biology Center, New York, NY USA; MRC Laboratory of Molecular Biology, United Kingdom

**Keywords:** cryoEM, automated cryoEM data collection, computer vision, microscope automation software, machine learning, deep learning, automation, single-particle cryoEM

## Abstract

Over the past decade, cryo-electron microscopy (cryoEM) has emerged as an important method for determining near-native, near-atomic resolution 3D structures of biological macromolecules. To meet the increasing demand for cryoEM, automated methods that improve throughput and efficiency of microscope operation are needed. Currently, the targeting algorithms provided by most data-collection software require time-consuming manual tuning of parameters for each grid, and, in some cases, operators must select targets completely manually. However, the development of fully automated targeting algorithms is non-trivial, because images often have low signal-to-noise ratios and optimal targeting strategies depend on a range of experimental parameters and macromolecule behaviors that vary between projects and collection sessions. To address this, *Ptolemy* provides a pipeline to automate low- and medium-magnification targeting using a suite of purpose-built computer vision and machine-learning algorithms, including mixture models, convolutional neural networks and U-Nets. Learned models in this pipeline are trained on a large set of images from real-world cryoEM data-collection sessions, labeled with locations selected by human operators. These models accurately detect and classify regions of interest in low- and medium-magnification images, and generalize to unseen sessions, as well as to images collected on different microscopes at another facility. This open-source, modular pipeline can be integrated with existing microscope control software to enable automation of cryoEM data collection and can serve as a foundation for future cryoEM automation software.

## Abstract 2

*Ptolemy* uses deep learning and computer vision to automate multi-scale targeting in cryoEM data collection. It accurately finds and ranks targets of interest (‘squares’ and ‘holes’) at successive magnification levels and generalizes across samples and microscopes.

## 1. Introduction

**Figure 1:**
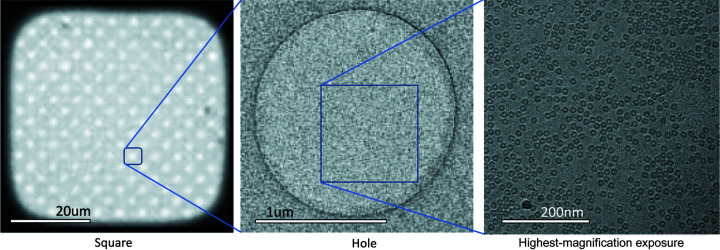
Example square, hole and highest-magnification exposure. Windows in the cryoEM grid containing ice are known as ‘squares’. Within squares are circular ‘holes’. Highest-magnification exposures are taken within holes. Ideal highest-magnification exposures contain particles (as shown) and have thin ice.

Cryo-electron microscopy (cryoEM) is a rapidly growing method for determining the structure of proteins in near-native conformations at high resolution (Bai *et al.*, 2015 ▸). CryoEM structure determination typically starts with the application of a solution containing purified protein to an EM grid, a holey substrate supported by a thin metal mesh (Cheng *et al.*, 2015 ▸). The sample droplet is then reduced to a thin liquid film and the grid is plunged into a cryogen, converting the thin film to a layer of vitrified ice (Egelman, 2016 ▸). The grid is then transferred to a transmission electron microscope (TEM) to collect high-magnification (high-mag) micrographs of the particles suspended in vitreous ice within the holes. Vitreous ice containing particles is found in windows in the grid termed ‘squares’ [Fig. 1 ▸(*a*)]. Within these squares are circular ‘holes’ [Fig. 1 ▸(*b*)] and particle images are obtained by taking high-resolution micrographs of the ice within these holes [Fig. 1 ▸(*c*)].

Each micrograph will typically provide numerous individual 2D projections of the protein particles, and these images can be processed to produce a three-dimensional map of the protein of interest (Wu & Lander, 2020 ▸). Solving a protein structure to high resolution usually requires tens to many hundreds of thousands of individual randomly oriented particle projection images which often requires collecting many thousands of high-quality high-resolution micrographs. Because EM grid preparation is not a well controlled process, the locations where highest-magnification data are to be collected must be identified from a series of successively increasing magnification images (Chua *et al.*, 2022 ▸).

**Figure 2:**
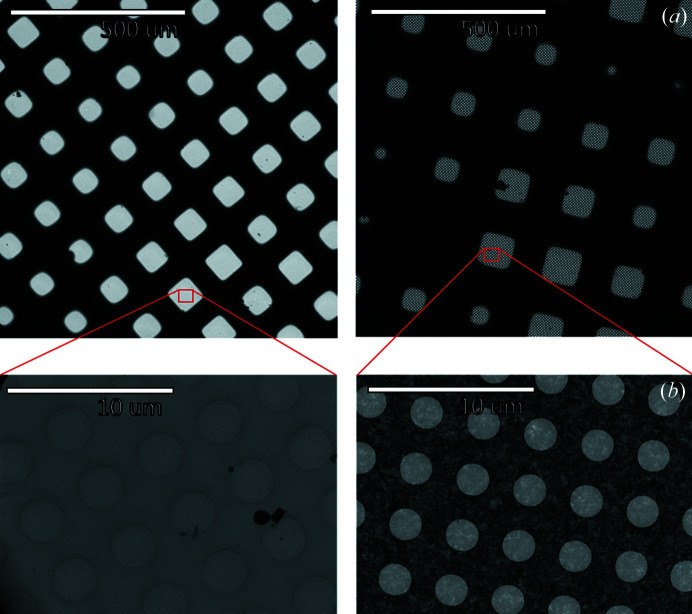
Example low- and medium-magnification images: carbon grid (left) and gold grid (right). First, low-magnification images of the grid, where squares are visible, are acquired at a pixel size of ∼200–500 nm pixel^−1^. Subsequently, medium-magnification images are acquired at a pixel size of ∼10–100 nm pixel^−1^ by imaging the regions inside the squares.

The process of collecting high-magnification data begins by taking low-magnification images of the grid [Fig. 2 ▸(*a*)], typically acquired at a pixel size of ∼200–500 nm pixel^−1^. Squares are selected from these images, and medium-magnification images [Fig. 2 ▸(*b*)] with a pixel size of ∼10–100 nm pixel^−1^ are taken within these squares. Holes and subsequent high-magnification collection locations are identified from the medium-magnification images. Not all squares or holes will be suitable for collection; the goal is to identify squares and holes in the grid with vitreous ice of suitable quality, ice that is the right thickness (typically slightly thicker than the largest diameter of the particle) and that contains a reasonable number of particles, ideally oriented in a range of angles (Noble *et al.*, 2018 ▸). Ultimately, the success of a data collection is determined by the quality of the resulting 3D reconstruction, which is a function of the number of particles found, the range of orientation angles present among the 2D projections, and the maximum resolution and signal-to-noise ratio (SNR) of the micrographs.

Automated data-collection software such as *Leginon* (Carragher *et al.*, 2000 ▸; Suloway *et al.*, 2005 ▸), *SerialEM* (Mastronarde, 2005 ▸) or *EPU* (made by ThermoFisher Scientific) provide several built-in tools for identifying potentially promising squares and holes. These tools include template-, correlation- or feature clustering-based image analysis algorithms and automated selection capabilities. However, none of these tools generalize out-of-the-box across the wide variety of grids that are encountered in practice. They can struggle to both detect holes and squares under contaminated or low-SNR conditions and to reliably prioritize good collection locations across different macromolecule specimens. This means that microscope operators must often manually identify squares in low-magnification images and tune parameters used for automated targeting of holes in medium-magnification images (https://em-learning.com). Additionally, existing hole targeting algorithms fail on a non-trivial percentage of cases, especially on noisy, contaminated or carbon grids with minimal contrast variation between the holes and substrate. Operators are then required to manually target holes in medium-magnification images which is a labor-intensive task. The human operator time required for targeting limits the efficiency of collection on expensive and over-subscribed cryo-transmission electron microscopes (cryo-TEMs). Additionally, with increasing detector speeds, automated targeting would allow for better utilization of microscope time that is otherwise wasted waiting for human input. Thus, fully automated targeting methods are needed to not only reduce the burden on human operators, but also to increase access and throughput for the entire scientific research community.

However, human-free automation of cryoEM data collection is challenging. Many types of EM grids exist, each with holes and squares of different shapes, sizes and spacings. The grids themselves are made from different materials (*e.g.* carbon or gold), which causes the resulting low- and medium-magnification images to have very different properties (Fig. S1 of the supporting information). Carbon grids, for example, have significantly less contrast between collection regions of interest (ROIs) in holes and background substrate at medium magnification compared with gold grids (Fig. S1). This is further complicated by variable sample preparation conditions leading to variable ice thickness and empty regions of the grid, along with deformations, contamination and lesions, all of which introduce visual artifacts (Fig. S1). In addition, cryoEM images have low SNR, especially at high magnification, and microscope parameters such as electron beam dose (often between 40 and 80 e^−^ Å^−2^) can significantly alter image properties (Cheng *et al.*, 2015 ▸). Furthermore, images at each magnification level may contain many collection ROIs or none at all (Lyumkis, 2019 ▸). Finally, even if good candidate collection ROIs are found, it is challenging to find ice containing many particles with enough diversity of projection orientations to produce high-quality 3D reconstructions.

In recent years, machine-learning techniques have transformed single-particle cryoEM data analysis. Tools such as *MicAssess* (Li *et al.*, 2020 ▸) and *MicrographCleaner* (Sanchez-Garcia *et al.*, 2020 ▸) allow for efficient post-processing of high-resolution micrographs collected, whereas others such as *Topaz* (Bepler *et al.*, 2019 ▸), *CASSPER* (George *et al.*, 2021 ▸), *Warp* (Tegunov & Cramer, 2019 ▸) and *crYOLO* (Wagner *et al.*, 2019 ▸) use deep learning to automate particle picking and image denoising. Machine-learning-based 3D reconstruction algorithms have also emerged, including *cryoSPARC* (Punjani *et al.*, 2017 ▸) and *CryoDRGN* (Zhong *et al.*, 2021 ▸). These methods have significantly improved our ability to analyze high-resolution cryoEM data quickly and thoroughly after collection. However, comparatively little attention has been given to accelerating or automating data collection itself. Yokoyama *et al.* (2020 ▸) recently introduced a machine-learning method for detection and classification of ROIs in medium-magnification images, but it requires retraining a model with an annotated medium-magnification image dataset for each data-collection session.

**Figure 3:**
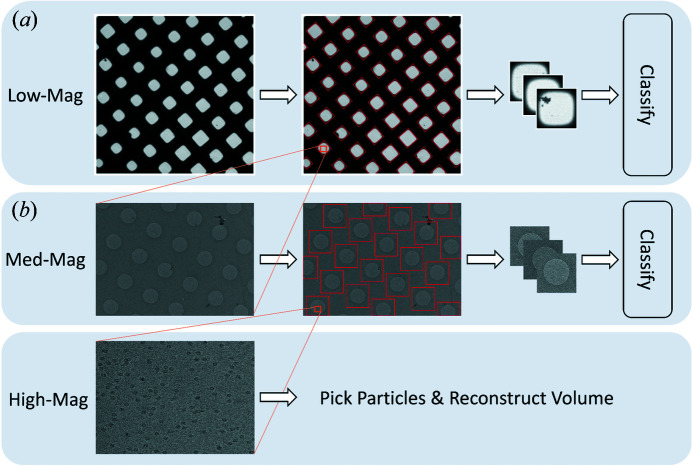
Pipeline overview. High-magnification images are taken from holes in medium-magnification images, which come from squares in low-magnification images. (*a*) *Ptolemy* detects, crops and then classifies squares in low-magnification images, which have a pixel size of approximately 200–500 nm pixel^−1^. (*b*) Next, Ptolemy detects, crops and then classifies holes in subsequent medium-magnification images, which have a pixel size of approximately 10–100 nm pixel^−1^.

To address these challenges in cryoEM data collection, we present *Ptolemy*, a pipeline that uses computer vision algorithms and pre-trained convolutional neural networks (CNNs) to navigate cryoEM grids at low and medium magnification and determine high-quality targeting locations without human input. We train the *Ptolemy* models on large datasets of low- and medium-magnification images with corresponding collection locations selected by operators from 55 different data-collection sessions. These sessions include carbon and gold holey grids and feature a variety of proteins, grid conditions, magnifications and electron beam dosages. Rather than attempting to learn separate models for different grid types or for different particles, we develop a single unified pipeline to localize and classify ROIs to approximate user selection locations in low- and medium-magnification cryoEM images (Fig. 3 ▸).

We demonstrate that *Ptolemy* can effectively detect and classify squares and holes in low- and medium-magnification images. We evaluate these predictions with comparison against operator-selected locations, while noting that operators target incompletely. We validate the models by holding-out entire data-collection sessions to confirm that the models generalize well to unseen sessions. Additionally, we compare our medium-magnification localization algorithm to an existing method (Yokoyama *et al.*, 2020 ▸) that performs medium-magnification localization (in that study, this is referred to as low-magnification localization) and show that our method yields superior generalization performance. Finally, a separate companion paper has been published: ‘*Fully automated multi-grid CryoEM screening using Smart Leginon*’, where the utility of *Ptolemy* in real-world collection cases is demonstrated and analyzed (Cheng *et al.*, 2022 ▸).

The *Ptolemy* source code is freely available for academic use at (https://github.com/SMLC-NYSBC/ptolemy) under CC BY-NC 4.0 license. *Ptolemy* is designed to be modular and to integrate directly with existing microscope control software. More information on *Ptolemy* can be found in Appendix *A* [App appa].

## 2. Methods

To automate microscope targeting for single-particle cryoEM data collection, we divide the problem into four sub-problems: (1) low-magnification square localization, (2) low-magnification square classification, (3) medium-magnification hole localization and (4) medium-magnification hole classification.

For low- and medium-magnification localization, the goal is to identify all possible collection ROIs: squares in low magnification and holes in medium magnification. The cropped ROIs are then fed into separate classification models at each level that determine whether these ROIs should be collected. Low-magnification localization is solved by pixelwise image segmentation using a mixture model, whereas medium-magnification localization is solved using a U-Net with a novel lattice-fitting algorithm (Gupta & Sortrakul, 1998 ▸; Ronneberger *et al.*, 2015 ▸). Classification at low magnification is achieved using a feedforward CNN whereas medium-magnification classification uses the U-Net localization probabilities because they outperformed a separate downstream classifier in our experiments. Training and hyperparameter information for all trained models can be found in Appendix *B* [App appb].

### 2.1. Datasets and splits

The data used to train and validate all models and algorithms come from 55 cryoEM data-collection sessions performed at the Simons Electron Microscopy Center (SEMC), a center within the New York Structural Biology Center (NYSBC), from 2018 to 2021. The sessions include gold and carbon grids, featuring regularly spaced and lacey holes, with tilted and untilted collection. Lacey-hole grids and tilted collection images were only used for low-magnification (square) localization and classification. Positive labels represent targeted selection locations used in these sessions. All labeled squares are manual operator selections on the low-magnification images. On the other hand, labeled holes on the medium-magnification images are generally automated selections optimized by operators using template-correlation-based hole localization and ice-thickness-based hole classification. A minority of labeled holes were also manually selected by operators. Data-collection sessions generally involve different samples, preparation methods and microscope settings (Weissenberger *et al.*, 2021 ▸). This results in considerable variation between sessions in the appearance of collection locations especially at medium magnification, as well as in the characteristics that make for good collection locations (Fig. S1). Therefore, to ensure that our models can generalize to unseen data-collection sessions with different experimental parameters, we primarily use session splits, where a set of sessions (termed ‘held-out sessions’) are withheld from the dataset used to train the models (training set). All performance metrics are reported from results on these held-out sessions.

### 2.2. Square localization

**Figure 4:**
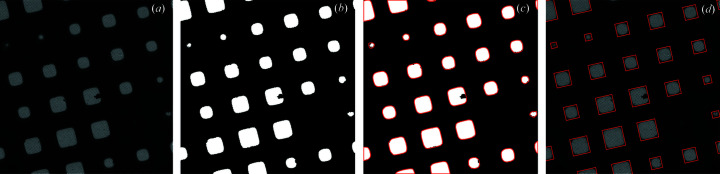
Example of the square localization procedure. (*a*) Original input image. (*b*) Mask recovered after segmenting pixels. (*c*) Finding convex polygons around the separate regions in the mask. (*d*) Aligned minimum bounding rectangles around the polygons, used for crops of the images.

The goal of square localization is to locate all squares (windows in the grid that may contain imageable ice) in low-magnification grid images. The input is a low-magnification image, and the output is a set of rectangular boxes tightly bounding the squares (Fig. 4 ▸). We find these boxes using a mixture model-based image segmentation algorithm followed by a geometric algorithm for identifying the aligned minimum bounding rectangles surrounding each square. Pixels in the image are first separated into two classes based on pixel intensity using a Poisson mixture model [Fig. 4 ▸(*b*)] (Forbes, 2018 ▸). Mixture model separation works because the distribution of pixels in the image can be accurately decomposed into low-intensity pixels coming from the thick grid bars in the surrounding background and higher-intensity pixels coming from the much thinner squares (Fig. S2). This approach avoids the need for a user to set a specific intensity threshold for identifying squares, which changes from session to session.

Next, we apply a flood filling algorithm to identify discrete regions from the segmented square pixels and then find a minimum bounding convex polygon to bound the pixels in each square [Fig. 4 ▸(*c*)]. Finally, we take advantage of the fact that the squares are axis-aligned to find the angle θ, for each low-magnification image where the minimum bounding rectangles aligned with θ bounding each minimum bounding convex polygon have the smallest total area. Formally, we seek 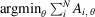 for *N* polygons, where *A*_*i*, θ_ is the area of the minimum bounding rectangle around the *i*th polygon, aligned at angle θ. We find this angle θ using bounded optimization (Brent, 1973 ▸), and the resulting minimum bounding rectangles are used to obtain aligned crops of the squares in the low-magnification image [Fig. 4 ▸(*d*)]. This algorithm is applied to 1304 low-magnification images, resulting in 41 000 crops of squares.

### 2.3. Hole localization

**Figure 5:**
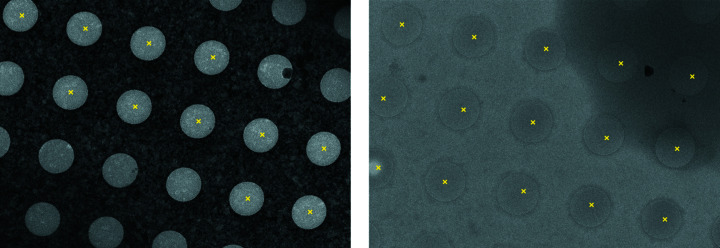
Example medium-magnification images. Human operator-selected locations are marked in yellow. The input to the U-Net is the image without any marking for the selection locations, and the output is a map with a 1 at each pixel where a selection was made and 0 elsewhere. In rare cases (right), holes can be darker than the surrounding region.

The goal of hole localization is to detect all hole locations in medium-magnification images. However, unlike in square localization, a mixture model-based segmentation approach does not work, because the difference in pixel intensities between the holes and the surrounding background is negligible, particularly for carbon grids. Some medium-magnification images even have ‘inverted’ holes where the pixel intensity within the hole is lower than the surrounding region (Fig. 5 ▸).

Here, our choice of model is informed by the available data. Although we do not have a dataset of bounding boxes around holes, we do have a large dataset of 28k carbon and gold holey grid medium-magnification images with locations at or near the center of holes where operators collected high-magnification micrographs. Therefore, we seek to learn the hole centers in each pixel-normalized medium-magnification input image by training a U-Net model to output a map with the same dimensions as the input containing 1 at the locations where the operator collected and 0 everywhere else (Figs. 5 ▸ and 6 ▸). We choose a U-Net architecture, because the neurons in the bottleneck layer have large receptive fields, allowing them to capture needed context, while the output layers use the information propagated from the bottleneck, as well as high-resolution features, to find the hole centers. The pixels in the medium-magnification image are normalized to control for variance in electron dose.

**Figure 7:**
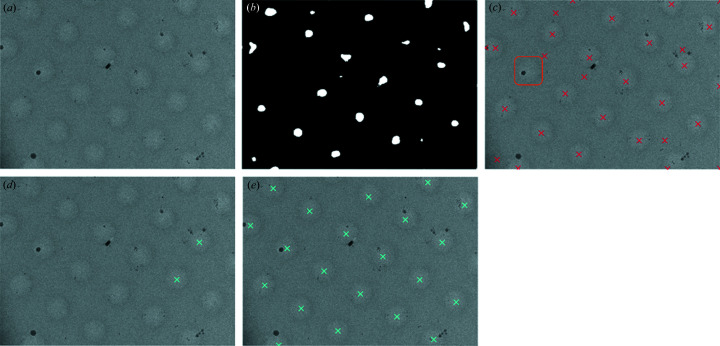
Example hole-center detection on a difficult image. On this low-contrast image, lattice fitting extends to missed holes while removing erroneous detections. (*a*) Input image. (*b*) U-Net output. (*c*) Centroids from high-probability regions in U-Net output (red). Many locations outside holes are detected incorrectly, and one hole (orange) is missed. (*d*) Running the optimal-lattice-finding algorithm results in finding lattice anchor points (cyan). (*e*) The lattice generated by these anchor points (cyan) results in coverage of all holes and cleans the incorrect detections.

Additionally, holes are known to lie on a regular square lattice, so we post-process the output of the U-Net to find the best fitting lattice. Given the lattice points in the image, we then crop around those points to extract hole images (Fig. 7 ▸). This helps to extend the predicted map from the U-Net to capture all holes in the image, not just the holes that the operators picked, while simultaneously cleaning erroneously detected regions. We find the lattice from the U-Net output map by searching pairs of candidate anchor points and selecting the pair for which the lattice produced by these anchor points has the smallest pixelwise error against the output map. We find

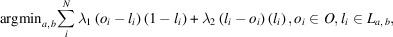

where *O* is the output of the U-Net, *N* is the number of pixels in the image, *L*_*a*, *b*_ is the lattice generated by anchor points *a* and *b*, and λ_1_ and λ_2_ allow us to independently weight the cost for false positives and false negatives. Candidate anchor point pairs are found by taking centroids of high-probability regions in the U-Net output map, and for each centroid, pairing with the *K* closest to other centroids. *K* trades performance for run time. Here we use *K* = 6.

Imposition of a regular square lattice over the U-Net output causes *Ptolemy* to be unable to handle tilted data collection because the tilt causes the hole centers to lie on a parallelogram-shaped (oblique) lattice rather than a regular square lattice. Support for tilted data collection will be added to a near-future update of *Ptolemy*.

To improve training, we apply Gaussian blur to the model output before computing the loss (Shorten & Khoshgoftaar, 2019 ▸). This helps, because the exact location the operator selects in a hole is noisy: the selection location that is near the center of the hole but there is often deviation from the exact center pixel, and the direction and magnitude of displacement from the center varies between medium-magnification images. Therefore, this smoothing allows the model to learn the centers of these holes, rather than having to learn the displacement from the center for every hole image. We also perform gradient descent on the sigma parameter of the Gaussian blur simultaneously with training the U-Net weights to allow the model to learn the optimal level of smoothing over training time (Fig. S3).

To improve generalization, we apply both random 90° rotation augmentation to the images during training as well as random inversion of the normalized pixels. Inversion of pixels is helpful, because for some sessions, particularly with carbon grids, the pixels in the holes are darker than the background pixels. Although pixel inversion augmentation allows for better carbon grid hole targeting, it does not affect gold grid images which do not suffer from contrast inversions.

### 2.4. Square and hole classification models

In square and hole classification, we aim to obtain rankings of squares and holes in images to prioritize the ordering with which they are targeted. Although there are many possible parameters that may be important for determining whether a square or hole contains high-quality particles, experienced operators are able to consistently find good locations, suggesting that at least some features of good target locations are identifiable in low- and medium-magnification images. Therefore, for each magnification we train a separate CNN to classify squares or holes as collected or not collected by operators. The input to our model is a cropped image of a square or hole, extracted using the square-localization method or hole-localization method above, and the output is a scalar probability.

#### 2.4.1. Square classification

We train our square classification model on a dataset containing 41k square crops, of which 11k were squares collected. Square images are normalized based on the intensity of all pixels within the bounding boxes for the squares in each low-magnification image to control for electron dose. We also include random forest (RF) and logistic regression (LR) models trained on summary statistics of the pixels extracted from the square image crops. This is because the operators typically use characteristics like the size/area and brightness of the squares to make their selections. Therefore, we include baselines which reflect this knowledge. The summary statistics used as features are mean intensity, maximum intensity, minimum intensity, variance in intensity, kurtosis, skew and crop area.

#### 2.4.2. Hole classification

We compare the summed pixelwise probabilities output by our localization U-net within each hole against two CNNs trained on a dataset containing 571k hole crops, of which 410k were targeted by operators. The dimensions of the holes and, therefore, the dimensions of the resulting crops vary widely between data-collection sessions. However, we do not want the model to use the size of the input image to decide if a hole is good or bad. We hypothesize that the location of image features within each hole (*e.g.* crystalline ice) is not important for classification. Rather, the presence, absence or proportion of these features is the main concern. Therefore, we compare between a standard CNN model that pads all input images to the same dimension and one which averages over non-channel dimensions of the final map before the fully connected layer, thereby treating the image as a bag of regions. Both CNNs normalize images based on pixels in the crop to control for difference in electron dose. Aside from the difference in padding versus average pooling, all other hyperparameters of the two models are identical.

## 3. Results and discussion

*Ptolemy* can accurately locate and rank collection locations within low- and medium-magnification images, with each stage producing good performance metrics, and results that appear reasonable on visual inspection. Furthermore, *Ptolemy* generalizes well to new sessions without user intervention or retraining on a session-by-session basis.

### 3.1. *Ptolemy* localization of squares in low-magnification images

**Figure 8:**
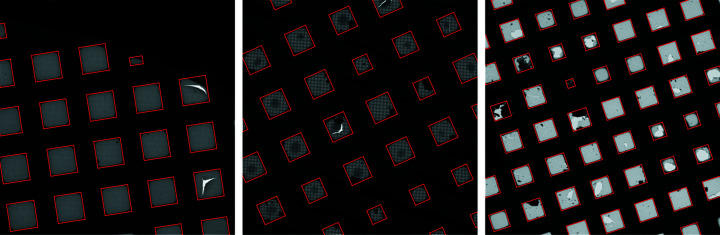
Example square localizations on low-magnification images. *Ptolemy* successfully segments bounding boxes around squares in the grid without human intervention.

The square localization algorithm successfully detects almost all operator-selected locations, as well as squares that were not collected, with few errors. Our algorithm successfully detects 98.8% of operator-selected locations (Table 1 ▸). An additional 30k unselected squares are detected, and visual inspection confirms that these are real squares that were not selected by the operator (Fig. 8 ▸).

### 3.2. Square classifier learns to rank squares effectively

For square classification, we explored three different models: an LR and RF on summary statistics (details in Appendix *C* [App appc]) extracted from square images, and a CNN on the images themselves. Both the RF and the CNN perform similarly on this task.

#### 3.2.1. Classifying squares for new sessions without prior knowledge

Square ranking without any information about the sample is a challenging task, but our models perform well and are significantly better than random guessing (Table 2 ▸). The task of ranking squares given the session split is particularly difficult, because the characteristics that make up good squares can vary from session to session. For optimum performance in this setting, a model would need to extract enough information from the square images to identify the optimal sample conditions and then predict the quality of the square accordingly. Although this is probably not possible for a classifier such as ours, which makes predictions for each square independently, we hypothesize that there are, at least, characteristics of squares that are always bad and maybe squares that are always good that can still provide useful guidance. The difficulty of this task is evident in comparison with the performance of the models on random splits of the data, where without the difficulty of generalizing to unseen sessions, the RF and CNN models perform significantly better (Table 2 ▸). Nonetheless, *Ptolemy* ranks squares significantly better than random guessing even in the session split setting.

**Figure 9:**
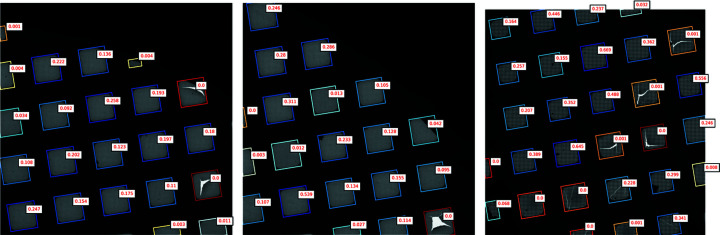
Example square classifications in low-magnification images. The model successfully prioritizes larger, brighter squares without cracks or contamination. Model-predicted probabilities for squares are in red. Colors from high to low score: dark blue, light blue, white, yellow, orange, red.

On visual inspection of predictions on held-out sessions, we find that the CNN makes reasonable predictions, with unbroken and larger squares prioritized over smaller, broken squares (Fig. 9 ▸). Additional example images with both model scores and user selection locations can be found in Fig. S4.

#### 3.2.2. Simple extracted features exhibit good performance on square classification

**Figure 10:**
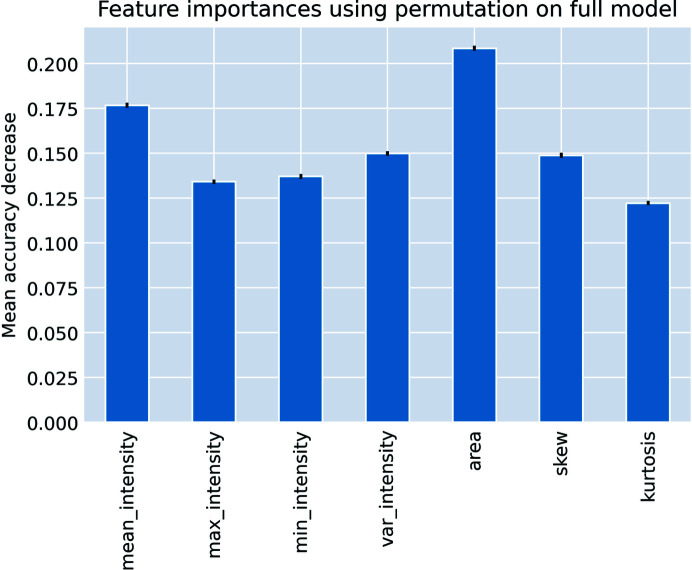
Feature importance for square classification. Results obtained for feature importance of square image summary statistics to predict whether a square is selected using an RF model. Area and mean pixel intensity are the most important features.

The RF performing comparably to the CNN on an image classification task is a surprising result for which there are several possible explanations. First, examining the feature importance of the extracted features for the RF models, using feature permutation, shows that area and mean pixel intensity are the most important features for predicting whether a square was selected (Fig. 10 ▸). This result aligns with our expectations, as operators usually use area and brightness of squares as primary criteria for selection. We hypothesize that the importance of area as a feature may partly explain the good performance of the RF relative to the CNN, as area may be a feature that is difficult for a CNN to learn. Additionally, since our dataset is not exhaustively labeled, a more complex CNN model may be learning redundant, irrelevant features that do not generalize well from training set to test set. Nevertheless, the CNN does not require a burdensome computational cost (it runs very quickly on commodity CPU hardware), and likely has a higher potential performance if a larger and cleaner dataset is curated. We therefore use it as the default model for *Ptolemy*.

### 3.3. *Ptolemy* retrieves more holes with fewer false positives

Next, we examine the performance of our methods for hole localization on medium-magnification images (Table 3 ▸). Since we do not have bounding box annotations for our dataset, we define a true positive as a model selection location that maps one-to-one with an operator-collected hole, a false negative as an operator-collected hole that contains zero or more than one (2+) model selection locations, and a false positive as a model selection location that is not contained in any operator-collected holes, or is only contained in operator-collected holes that also contain other model selection locations. For more information on how we defined ‘model selection locations’ for each model in Table 3 ▸, see Appendix *D* [App appd].

We find our U-Net, without lattice fitting, can learn operator-selected locations exceptionally well, and can identify 98.4% of all operator-selected holes with 70.3% precision (Table 3 ▸, row 2). We compare this method with the Yolov5-based model trained by Yokoyama *et al.* (2020 ▸) on the same dataset and find that the U-Net is superior in both recall and precision (Redmon & Farhadi, 2018 ▸).

#### 3.3.1. Lattice fitting reduces false negative rate

**Figure 11:**
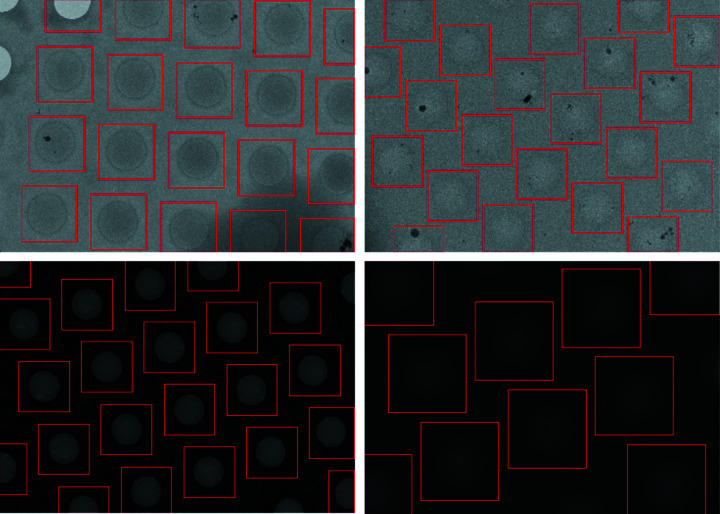
Examples of hole localization using the U-Net + Lattice Fitting. *Ptolemy* successfully detects all holes across a wide range of hole sizes, brightness and contrast conditions, from easily visible gold grids (bottom left) to very low contrast carbon grids that are difficult to see even for humans (top right).

With the addition of lattice fitting (Table 3 ▸, row 3), we reduce the false negative rate by a factor of 2, from 1.6% to 0.7%. Although the precision is also reduced with lattice fitting, we are primarily concerned with improving the false negative rate (1-recall) as our aim is to recover all the holes at this stage. In the manual inspection, we find that the U-Net with lattice fitting correctly locates holes across diverse images (Fig. 11 ▸). Because many holes are not selected by the operators, we expect that, for the goal of detecting all holes (selected and not selected), lattice fitting is helpful. Additionally, only keeping lattice tiles based on a probability taken from the U-Net output (Table 3, ▸ row 4) significantly improves precision at the cost of recall.

#### 3.3.2. U-Net + Lattice Fitting generalizes hole localization to an external dataset

To further test generalization, we predict holes in the dataset by Yokoyama *et al.* (2020 ▸) using our U-Net + Lattice Fitting model. Yokoyama *et al.* (2020 ▸) reported 95–97% recall of their own Yolov5 based model on these data. We find that the U-Net + Lattice Fitting model generalizes well, achieving 0.685 precision, 0.950 recall and 0.796 F1 score. The images in this dataset were collected from an external facility, using a different microscope (*Ptolemy* data were collected primarily on a TFS Krios and a Glacios; this dataset was collected on a JEM-Z300CF) and with different magnification compared with the images in our training dataset (Yokoyama *et al.*, 2020 ▸), demonstrating that *Ptolemy* generalizes effectively.

### 3.4. *Ptolemy *successfully classifies holes in medium-magnification images after localization

**Figure 12:**
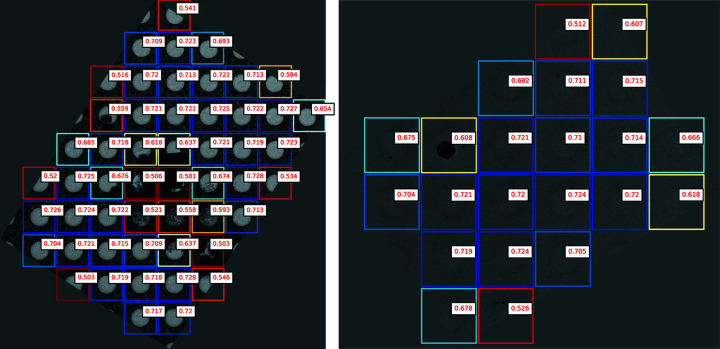
Example hole classifications in medium-magnification images using the average-pooling model. *Ptolemy* can successfully prioritize complete holes free of contaminants. Model-predicted probabilities for holes are in red. Colors from high to low score: dark blue, light blue, white, yellow, orange, red.

Next, we examine the performance of our hole classification models (Table 4 ▸). Both the padded model and the average-pool model perform well on the hole classification task. In example images, we see that the model can effectively separate good, unblemished holes from those with blemishes and artifacts on both gold and carbon grids (Fig. 12 ▸).

#### 3.4.1. Average-pooling improves hole classification

The average-pool model slightly outperforms the padded model, which supports our hypothesis that the location in the image where features occur is not as important in determining hole quality, and that given the wide variance of hole sizes, a model that uses average pooling is preferable to padding all crops to the same size.

#### 3.4.2. Classifying holes using the localization U-Net output

We compare the dedicated CNN classifiers against the sum of the hole localization U-Net probabilities within a crop to determine the probability of picking a hole (Table 4 ▸, row 3). Surprisingly, the U-Net score outperforms the dedicated CNN classifiers in accuracy and ROC AUC. This is probably because the U-Net uses the context around the hole to help predict where the hole was collected from. The U-Net is deep to allow large holes to fit entirely within the receptive field of the bottleneck-layer neurons. This means that, for grids with smaller holes, the U-Net will have information about the location of the hole on the grid and the characteristics of nearby holes that our classifiers, which use only hole crops, lack.

### 3.5. Training and evaluating human operator selections

One of the major challenges in developing *Ptolemy* is the lack of fully annotated data for training and assessment of model performance. We rely on training data composed of incomplete expert operator selections. These selections only represent an expert guess at the best collection locations and our models are trained to recapitulate these operator selection decisions as a surrogate for selecting high-quality data. Ideally, we would train and evaluate our models based on the true end goal of cryoEM data collection, particle quantity and quality, as determined by the resulting 3D structures, but these data are currently unavailable.

Furthermore, the operators do not exhaustively select all possible viable collection locations. Since our model evaluation considers locations that the operator did not select as ground-truth negatives, the reported precision values are likely to underestimate the true precision of *Ptolemy* models. This lack of exhaustive selection also creates biases in the training data, especially on the hole and square classification tasks, which may be learned by our models. For example, due to the microscope setup at the SEMC/NYSBC, holes near the edge of medium-magnification images are often not collected even though they may be viable collection locations.

### 3.6. Future work

A large fraction (the bulk majority at NYSBC) of cryoEM data-collection experiments involve gold or carbon holey grids generated via blotting and collected without tilting. Development, training and validation of *Ptolemy* were carried out with the goal of providing a system for automated collection on these grids. However, this leaves a long tail of less used but significant grid types and preparation methods for which future work is required. These include lacy grids, grids generated via spray or spotting methods, and tilted collection. Furthermore, *Ptolemy* currently lacks the ability to update its predictions dynamically to adjust to a given collection session. *Ptolemy* is designed to allow for modular improvement over time, so future updates of *Ptolemy* can build on the current set of algorithms to cover these cases as well. Tilted images can be corrected with basic image processing, as the angle of the tilt will be known in advance, and then the un-tilted images can be processed as normal. Grids generated by spray or spotting methods may need new purpose-built algorithms, but they may also be effectively handled by an on-the-fly learning algorithm. Lacy grids will likely require the most customization, including separate segmentation and prioritization algorithms at the medium-magnification level.

## 4. Conclusions

Increasing throughput and reducing cost through automation is necessary to meet increasing demand for cryoEM. In this work, we present *Ptolemy*, an open-source, modular package for automatic targeting and classification of cryoEM low- and medium-magnification images using purpose-designed computer vision and deep-learning algorithms. *Ptolemy* accurately localizes and ranks squares and holes in low- and medium-magnification images across a wide range of image and sample conditions. By training on large datasets of sparse microscope operator selection locations, the *Ptolemy* localization algorithms generalize to diverse gold and carbon holey grids and rank potential collection locations effectively without session-specific parameters, as we have demonstrated on held-out collection sessions within the SEMC/NYSBC dataset and on an independent dataset from another facility. Additionally, *Ptolemy* has been integrated with the microscope control software *Leginon* to enable automated data collection in real-life use cases (Cheng *et al.*, 2022 ▸).

*Ptolemy* is similar to the recent works *SmartScope* (Bouvette *et al.*, 2022 ▸) and *CryoRL* (Li *et al.*, 2022 ▸), which both offer automated collection capabilities. *SmartScope* focuses on automated screening and providing a helpful user interface to view and control screening in real time, whereas *CryoRL* focuses on optimizing the cryoEM data collection as a path-planning problem and does not clearly address the square- and hole-detection problem. Both primarily repurpose existing deep-learning object detection and classification algorithms. *Ptolemy*, on the other hand, uses novel algorithms that are purpose-built for detection and classification of holes and squares in cryoEM images.

Although *Ptolemy* identifies and generally ranks ROIs remarkably well, it is unable to incorporate session-specific information to reprioritize targets on-the-fly. In future work, we plan to use the current *Ptolemy* classification models as prior models, and dynamically update these prior models during each collection session based on the quality of highest-magnification exposures that are collected from explored squares and holes in an active-learning framework.

*Ptolemy* is a significant advance in the automation of cryoEM data collection, allowing for fully unattended data collection, and increasing microscope and operator efficiency. To accelerate cryoEM collection for the whole community, *Ptolemy* is open source and freely available for academic use at https://github.com/SMLC-NYSBC/ptolemy. We anticipate that *Ptolemy* will become an integral part of the data-collection pipeline and will serve as the basis for future work in cryoEM automation.

The supporting information file pw5021sup1.xlsx contains the metadata for the various data-collection sessions used to train and test the models in *Ptolemy*.

## Supplementary Material

Metadata about the sessions used. DOI: 10.1107/S2052252522010612/pw5021sup1.xlsx

Supporting figures. DOI: 10.1107/S2052252522010612/pw5021sup2.pdf

## Figures and Tables

**Figure 6:**
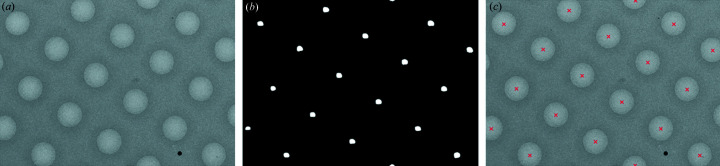
Example hole-center detection without lattice fitting. For some cases, taking the centroids of the high-probability regions predicted by the U-Net is sufficient. (*a*) Input image. (*b*) U-Net output. (*c*) Centroids from high-probability regions in U-Net output (red).

**Table 1:** Statistics from running the square localization procedure on low-magnification images

No. of operator-selected locations	10993
No. of operator-selected locations detected	10857
Total no. of locations detected	41301

**Table 2:** Performance metrics of different ML models on the square classification task measured on held-out sessions and held-out square images We compare the performance of LR, an RF and a CNN based on two metrics. ROC AUC: area under receiver-operating characteristic curve.

	Session split		Random split	
Model	ROC AUC	Average precision	ROC AUC	Average precision
LR	0.539	0.258	0.499	0.259
RF–5 feature	0.603	0.344	0.867	0.734
CNN	0.608	0.331	0.733	0.489

**Table 3:** Performance metrics of different methods on held-out sessions for hole localization from medium-magnification images Reported metrics are aggregated by session and averaged. Definitions of true positive, false positive and false negative used for computing precision, recall, and F1 can be found in Section 3.3 and Appendix *D* [App appd].

Model	Precision	Recall	F1
Yolov5 (from Yokoyama *et al.*, 2020 ▸)	0.395	0.669	0.459
U-Net	0.703	0.984	0.815
U-Net + Lattice Fitting	0.549	0.993	0.702
U-Net + Lattice Fitting + Probability Threshold	0.802	0.891	0.837

**Table 4:** Performance of hole classification CNNs on hold-out sessions ROC AUC: area under receiver operating characteristic curve.

Model	Accuracy	ROC AUC	Average precision
CNN (padding)	0.748	0.742	0.808
CNN (average pool)	0.758	0.796	0.878
U-Net + Probability Threshold	0.846	0.868	0.867

## Appendix A *Ptolemy* implementation details and default models

*Ptolemy* is an operating system and microscope control software agnostic and requires only Python (3+) and a few installed packages to run. Package dependencies can be found in the github repository at https://github.com/SMLC-NYSBC/ptolemy. By default, *Ptolemy* is configured to use the mixture model for low-magnification square detection, the CNN for low-magnification square classification, the U-Net with grid fitting for medium-magnification hole detection and the average-pooled CNN for medium-magnification hole classification. It runs fully on CPU and requires a maximum of only 2.7 GB RAM to detect and classify squares in a low-magnification image and 3.6 GB RAM to detect and classify holes in a medium-magnification image.

After installation, running *Ptolemy* during data collection involves the following steps.

(1) Microscope control software captures tiled low-magnification mrc(s) of the grid and saves the file.

(2) Call python lowmag_cli.py (path to low-magnification mrc). This returns a JSON containing a list of dictionaries with the fields (i) vertices corresponding to vertex coordinates of squares, (ii) center corresponding to the centers of squares, (iii) area corresponding to areas of squares, (iv) brightness corresponding to mean brightness of pixels in squares, (v) score corresponding to CNN predicted score (between 0 and 1) of squares.

(3) Use the output coordinates and scores to select the collection location(s) to collect medium-magnification images.

(4) Microscope control software captures medium-magnification mrc(s) within squares and saves to file.

(5) Call python medmag_cli.py (path to medium-magnification mrc). This returns a JSON containing a list of dictionaries with the fields (i) vertices corresponding to vertex coordinates of hole crops, (ii) center corresponding to the centers of holes, (iii) area corresponding to areas of hole crops, (iv) score corresponding to CNN predicted score (between 0 and 1) of holes.

(6) Use the output coordinates and scores to select the collection location(s) to collect the highest-magnification exposures.

(7) Return to step (3) to explore new medium-magnification collection locations from existing low-magnification images, or step (1) to select new low-magnification collection locations.

For an example of integrating *Ptolemy* into cryoEM data-collection software in practice, please refer to the companion paper to *Ptolemy*, ‘*Fully automated multi-grid CryoEM screening using Smart Leginon*’ (Cheng *et al.*, 2022 ▸).

## Appendix B Training and hyperparameters

All deep-learning and machine-learning models were trained using default hyperparameters in *PyTorch* (Paszke *et al.*, 2019 ▸) and *scikit-learn* (Pedregosa *et al.*, 2011 ▸) except where stated otherwise. All deep-learning model parameters were fitted with the default *Adam*, except for the sigma parameter of the Gaussian smoothing, which was initialized to *e* and trained with an *Adam* optimizer with a learning rate of 0.1 (Kingma & Ba, 2017 ▸). Binary-cross-entropy loss was used for all deep-learning models, and for the U-Net a positive weight of 100 was applied.

The square classification CNN was trained for two epochs and used two 5 × 5 convolutional layers followed by three 3 × 3 convolutional layers, with 64 channels per layer and with a batch size of 128, whereas the hole classification CNN was trained for five epochs and used one 5 × 5 convolutional layer, followed by three 3 × 3 convolutional layers with 128 channels per layer, with a batch size of 32. Both models used batch normalization, max-pooling and ReLU activations (LeCun *et al.*, 2015 ▸; Ioffe & Szegedy, 2015 ▸).

The U-Net for hole localization was trained for 6000 steps and used nine down-blocks and nine up-blocks, with each down-block and up-block using a 64 channel 3 × 3 kernel convolutional layer. The model also used ReLU activations for down- and up-blocks, max-pooling for downsampling in down-blocks, and nearest interpolation for upsampling in up-blocks. Batch norm was not used. Also, the bias of the final convolutional layer which produces the final output of the model was initialized at −10 to allow the model to initially predict all or mostly zeros in the output, since the target image contains zeros everywhere except for the few pixel locations where the operator made a selection.

Random 90° rotation augmentation is applied while training square and hole classification CNNs. Random 90° rotation augmentation is combined with random pixel inversion when training the U-Net for hole localization.

## Appendix C Random forest and logistic regression model details

The RF and LR models were trained on the following features for the squares: mean pixel intensity, maximum pixel intensity, minimum pixel intensity, variance of pixel intensities, skew of pixel intensities, kurtosis of pixel intensities and area. Default hyperparameters from *scikit-learn* were used for both models.

## Appendix D Definition of ‘predicted collected regions’ for each model

For U-Net + Lattice Fitting, crops are generated by creating squares centered at each lattice point with a side length equal to *d*_l_ − 60, where *d*_l_ is the distance between lattice points. All crops are considered predicted collected regions.

For U-Net alone, circles with a radius of 50 pixels around each centroid of high-probability regions in the U-Net output map are considered predicted collected regions.

For U-Net + Lattice Fitting + Probability Threshold, we generated crops like in U-Net + Lattice Fitting, but then only kept the crops where the sum of pixel probabilities outputted by the U-Net within the crop was greater than 0.5.

For the Yolov5 model in Yoneo-Locr, which outputs many bounding boxes at different confidence levels, we had to decide how to set confidence thresholds which determine the bounding boxes that are kept. We aimed to be generous to the model by picking the confidence threshold that gave the maximum F1 score for each image independently and keeping all bounding boxes in that image that were predicted with confidence greater than this threshold.
