# Supplementary material for: Learning to automate cryo-electron microscopy data collection with Ptolemy
Source: IUCrJ. 2023 Jan 1;10(Pt 1):90–102. doi: 10.1107/S2052252522010612 (PMC9812219; doi:10.1107/S2052252522010612)
Supplement: Supplementary file 2 [file m-10-00090-sup2.pdf]

IUCrJ

Volume 10 (2023)

Supporting information for article:

Learning to automate cryo-electron microscopy data collection with Ptolemy

Paul T. Kim, Alex J. Noble, Anchi Cheng and Tristan Bepler

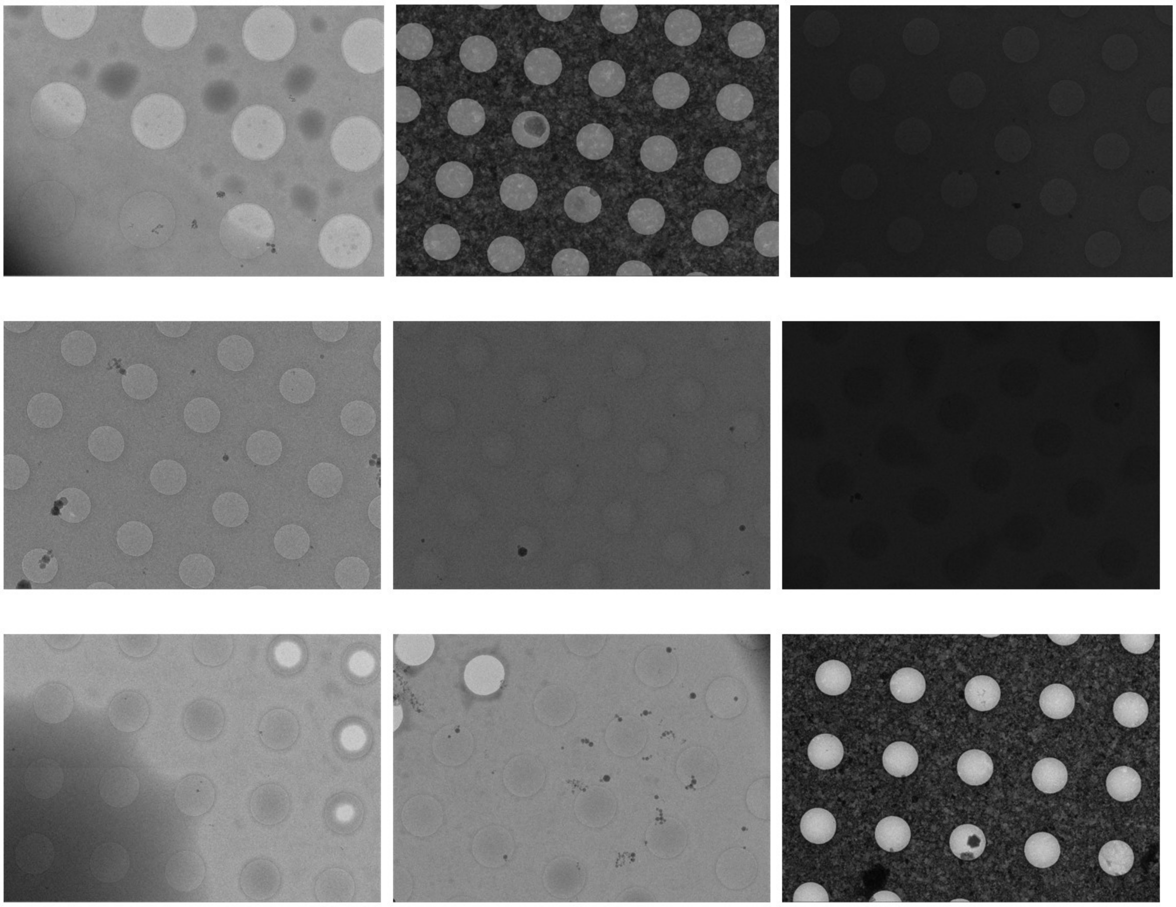

Figure S1 Example medium-magnification images showing variance present in grid types (gold, carbon), spacing (ranging from 1-100μm) and hole size (ranging from 0.6-17.5μm). Second and last images feature gold grids, while the remaining feature carbon grids of varying difficulty.

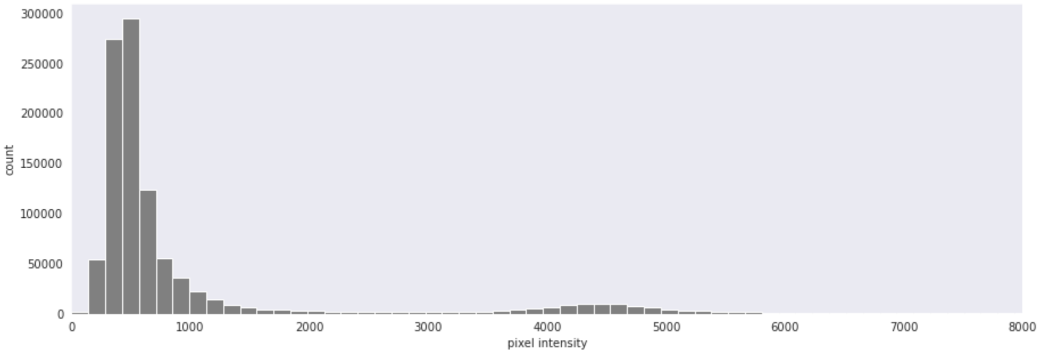

Figure S2 Histogram of pixel intensities for an example low-magnification image. The pixel intensities from the squares and grid bars decompose into two separate distributions with little overlap, thereby allowing a mixture model to separate pixels into two classes.

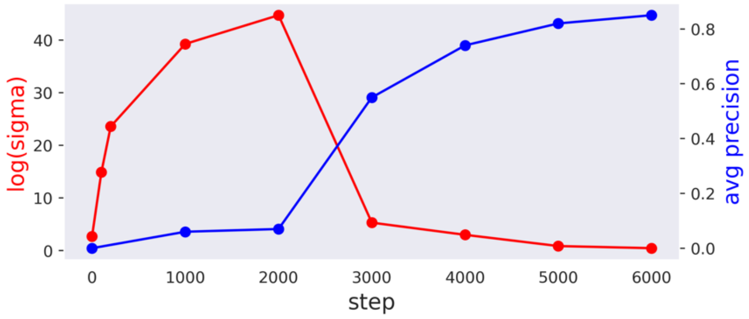

Figure S3 Sigma parameters versus model training progress. We plot the Gaussian smoothing sigma parameter against average precision on the validation set during training of the U-Net. Initially, sigma increases as the U-Net poorly reproduces the operator-selected locations but then sigma falls as the U-Net learns to identify hole centers better and only needs a small amount of smoothing to account for the uncertainty in the exact location that the operator-selected near the center of the hole.

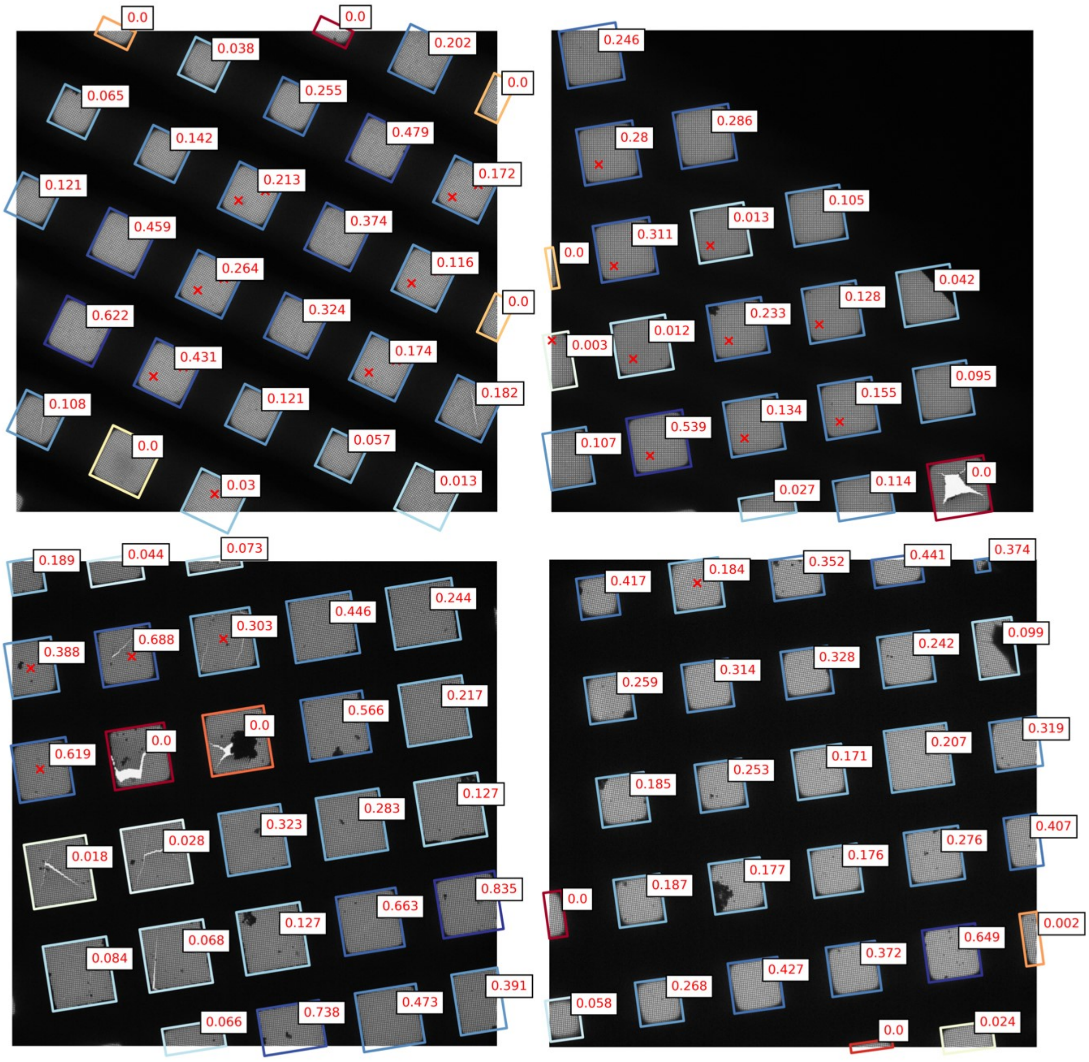

Figure S4 Example images of model predictions and user selections for square selection task. Operator selections located at red x.
